# The proportion of tuberculosis disease among adult patients receiving biologic agents at a tertiary centre in Kenya

**DOI:** 10.1016/j.ero.2026.100263

**Published:** 2026-10-26

**Authors:** Subo Hassan Marsa, Fredrick Otieno Owuor, Jasmit Shah, Felix Riunga Mutuma

**Affiliations:** 1 Department of Internal Medicine, Aga Khan University, Nairobi, Kenya; 2 Brain and Mind Institute, Aga Khan University, Nairobi, Kenya

## Abstract

**Objectives:**

Tuberculosis (TB) remains a major global health challenge. Kenya is a high-TB burden country, with a prevalence of 558 cases per 100,000 adults according to the 2016 Kenya Tuberculosis Prevalence Survey. Biologic agents have been linked to an increased risk of TB infection due to their immunosuppressive effects. However, data on TB occurrence among patients receiving biologic agents in Kenya are lacking. This study aimed to determine the proportion of TB disease among adult patients receiving biologic agents at a tertiary centre in Kenya.

**Methods:**

A retrospective cohort study of adult patients receiving biologic agents at Aga Khan University Hospital, Nairobi, between January 2018 and December 2023 was conducted. Data were extracted from the medical records of eligible patients using an abstraction tool, and statistical analysis was performed.

**Results:**

A total of 204 patients were included. The median age was 45.8 years (IQR, 36.3-60.1), and 59.3% were female. Rheumatologic disorders were the most common indication for biologic therapy, followed by malignancies and neuroinflammatory disorders. Anti-CD20 agents, predominantly rituximab, accounted for 71.6% of biologic use. Only 69.1% of patients had pretreatment TB infection screening. Three patients (1.5%) developed TB disease, all while receiving rituximab.

**Conclusions:**

All TB disease cases occurred during rituximab therapy, contrary to evidence emphasising a higher risk with anti-tumour necrosis factor alpha agents, highlighting the importance of systematic TB screening and monitoring across all biologic therapies in TB-endemic settings.

**WHAT IS ALREADY KNOWN ON THIS TOPIC**

 

• Biologic therapies have transformed the management of autoimmune and inflammatory diseases but are associated with an increased risk of opportunistic infections, particularly tuberculosis (TB). While the relationship between biologic therapy and TB has been extensively studied in Europe and North America, there remains limited evidence from high-TB burden regions in sub-Saharan Africa.

**WHAT THIS STUDY ADDS**

 

• To our knowledge, this is the first study on TB disease outcomes among biologic-treated patients in Kenya and, more broadly, East Africa, looking at the detailed characterisation of biologic therapy exposure across multiple specialities and indications. The findings provide clinically relevant information regarding TB risk, TB infection screening practices, and biologic prescribing patterns in a high-TB burden setting. Interestingly, in this retrospective cohort study, all TB cases occurred in patients receiving rituximab, with none noted in tumour necrosis factor alpha (TNF-α) inhibitor and interleukin-6 inhibitor users, indicating that TB risk may extend beyond TNF-α inhibitors in TB-endemic settings.

**HOW THIS STUDY MIGHT AFFECT RESEARCH, PRACTICE OR POLICY**

 

• We believe that this study will be of interest to readers of *EULAR Rheumatology Open*, particularly clinicians managing patients receiving biologic therapies in TB-endemic regions where data on biologic therapy safety remain limited and encourage further prospective multicentre studies with larger and more diverse populations to better inform context-specific screening and prevention strategies.

Alt-text: Unlabelled box dummy alt text

## INTRODUCTION

Tuberculosis (TB) remains a major global public health challenge, with a disproportionate burden in low- and middle-income countries, particularly in sub-Saharan Africa (SSA) [1]. In 2024, an estimated 10.7 million individuals developed TB and 1.23 million died from the disease, underscoring its persistent morbidity and mortality worldwide [2]. Kenya is among the high-TB burden countries, where transmission is driven by factors such as HIV coinfection, inadequate healthcare infrastructure, and other socioeconomic determinants [[3], [4], [5], [6]]

The introduction of biologic agents has significantly improved outcomes in autoimmune and inflammatory diseases, including rheumatoid arthritis, spondyloarthritis, and other systemic conditions. These therapies target specific immune pathways, such as tumour necrosis factor alpha (TNF-α), interleukin-6 (IL-6), and B cells [7,8]. However, their immunosuppressive effects increase susceptibility to opportunistic infections, particularly TB [[9], [10], [11], [12], [13]].

Among these agents, TNF-α inhibitors are well established to increase the risk of reactivating TB infection. TNF-α plays a critical role in maintaining granuloma integrity and controlling Mycobacterium tuberculosis infection; its inhibition disrupts this defence mechanism, increasing the risk of progression to TB disease [12,[14], [15], [16]]. Consequently, international guidelines recommend routine TB infection screening before initiating biologic therapy, typically using tuberculin skin tests (TSTs) or interferon-gamma release assays (IGRAs) [17,18].

Despite these recommendations, most available data on TB risk in patients receiving biologic therapy originate from high-income, low-incidence settings. Evidence from TB-endemic regions, including SSA, remains limited. This gap is particularly crucial given the higher baseline prevalence of TB infection and the potential limitations of current screening tools in immunocompromised populations [17,19].

This study aimed to determine the proportion of TB disease among adult patients receiving biologic agents at a tertiary referral centre in Nairobi, Kenya. Additionally, it evaluated the patterns of TB disease manifestation, timing of onset relative to biologic therapy, and the uptake and methods of TB infection screening in this vulnerable population.

## MATERIALS AND METHODS

### Study setting and design

This was a retrospective cohort study conducted at Aga Khan University Hospital, Nairobi, Kenya (AKUHN), a private tertiary referral centre serving a diverse patient population in Kenya. Medical records of adult patients receiving biologic agents between January 1, 2018 and December 31, 2023 were reviewed.

### Study population

Eligible participants included all adult patients managed using biologic agents at the AKUHN, irrespective of the therapy type or indication, and meeting inclusion criteria during the study period.

#### Inclusion criteria

a. Age ≥ 18 years.
b. Initiated on a biologic agent at the facility from January 1, 2018 and followed up for at least 3 years post therapy.

#### Exclusion criteria

a. Patients with known TB disease before starting a biologic agent.
b. Those admitted to the facility while already on a biologic agent.
c. Patients lost to follow-up before 3 years post initiation of biologic treatment.
d. Any medical record with missing or incomplete data.
e. Demise before 3-year follow-up unrelated to TB disease.

Patients who initiated biologic agents prior to January 1, 2018 were excluded even if their subsequent follow-up extended into the study period. This cut-off was applied to standardise the cohort’s index date of treatment initiation across the full observation window, ensuring uniform ascertainment of baseline characteristics, pretreatment TB screening practices, and dosing data from a consistent starting point, and to avoid left-truncation bias that would arise from including patients with variable, undefined durations of prestudy biologic exposure of unknown TB-risk relevance.

### Data collection

Data were extracted using a standardised abstraction tool and entered into REDCap (Research Electronic Data Capture) software from electronic health records and medical files. Variables collected included demographic characteristics including biological sex(categorized as male or female as recorded in patients medical records), underlying diagnoses, biologic agent type and duration, TB infection screening methods, occurrence and type of TB disease, diagnostic modalities, and relevant comorbidities, including HIV status and prior TB history. Indications for biologic therapy were categorised into rheumatologic, dermatologic, neuroinflammatory, oncologic, and other conditions.

### Definitions

TB disease was defined by microbiological confirmation (culture for M. tuberculosis, GeneXpert/PCR, or acid-fast bacilli smear), histological or radiological findings consistent with TB, or clinical diagnosis supported by symptoms (fever, weight loss, and night sweats) and initiation of anti-TB therapy.

TB infection was defined by a positive IGRA or TST in the absence of clinical or radiological evidence of TB disease. Screening practices were based on international recommendations, as no standardised national protocol exists.

### Data analysis

Continuous data were presented as medians and IQRs, whereas categorical data were presented as frequencies and percentages. Group comparisons were performed using the Kruskal-Wallis test for continuous data and Fisher’s exact test for categorical data. A *P* value < .05 was considered statistically significant. Analyses were conducted using IBM SPSS Statistics for Windows, version 23.0 (IBM Corp. , NY,USA).

### Ethical considerations

Ethical approval was obtained from the Aga Khan University Institutional Scientific and Research Ethics Committee (ISERC) (Ref: 2024/ISERC-126 [v2]) and a research licence from the National Commission for Science, Technology, and Innovation (NACOSTI/P/25/4172542). A waiver of informed consent was granted due to the retrospective nature of the study. Patient confidentiality was maintained through data deidentification and secure storage in a password-protected REDCap database accessible only to the investigators.

## RESULTS

### Study population

A total of 939 patients who received biologic agents between January 2018 and December 2023 at AKUHN, were identified. Of these, 735 were excluded due to incomplete records (62), loss to follow-up (509), prior TB disease (3), demise before 3-year follow-up unrelated to TB (147), or biologic initiation outside the study period (14). The final analysis included 204 patients. Figure summarises the study population.

**Figure:**
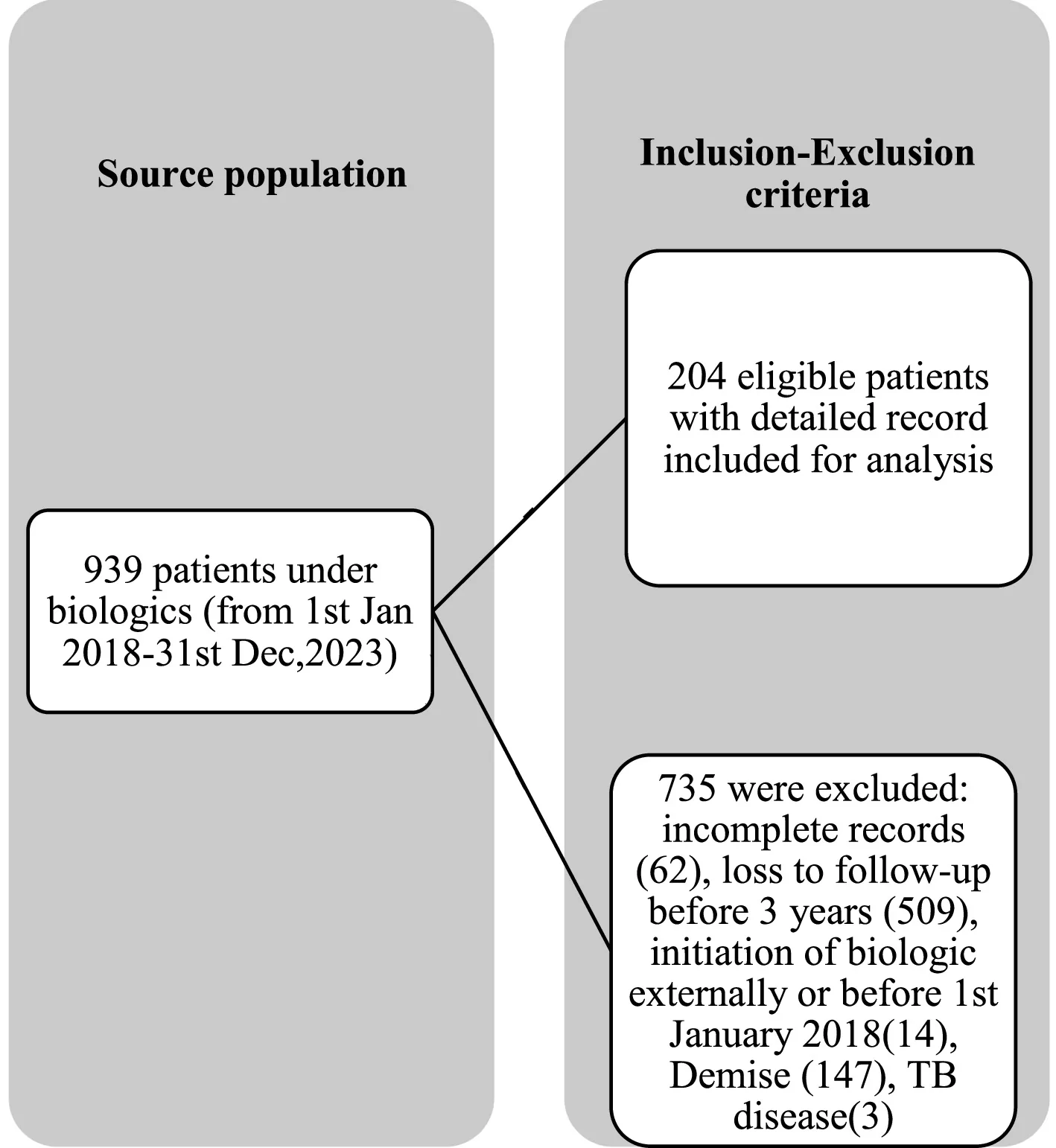
Flowchart of the selection of the study population.

### Demographic and clinical characteristics of the study population

The median age was 45.8 years (IQR, 36.3-60.1), and 59.3% were female. Rheumatologic disorders were the most common indication for biologic therapy (30.9%), followed by malignancy (21.1%) and neuroinflammatory disorders (18.1%). The most frequently used biologic class was anti-CD20 therapy (71.6%), predominantly rituximab. HIV status was negative in 62.7% of patients, although undocumented in 33.8%. Among documented comorbidities, hypertension and diabetes mellitus were most prevalent. Table 1 describes the demographic and clinical characteristics of the study population.

**Table 1:** Demographic and clinical characteristics of the study population

Age (y) (median [IQR])	45.8 [36.3, 60.1]
Sex	
Female	121 (59.3%)
Male	83 (40.7%)
Primary diagnosis category	
Rheumatologic disorder	63 (30.9%)
Neuroinflammatory disorder	37 (18.1%)
Malignancy	43 (21.1%)
Other	61 (29.9%)
Biologic agent class	
Anti-CD-20	146 (71.6%)
IL-6 inhibitors	41 (20.1%)
Anti-TNF alpha	19 (9.3%)
Biologic agents	
Rituximab	138 (94.5%)
Ocrelizumab	11 (7.5%)
Obinutuzumab	2 (1.4%)
Tocilizumab	41 (100%)
Golimumab	15 (78.9%)
Infliximab	4 (21.1%)
HIV status	
Negative	128 (62.7%)
Positive	7 (3.4%)
Unknown	69 (33.8%)
Comorbidities (n = 22)	
Diabetes mellitus	9 (40.9%)
Hypertension	11 (50.0%)
Chronic kidney disease	5 (22.7%)
Other	8 (36.4%)
Other comorbidities (n = 8)	
Bullous pemphigoid	1 (12.5%)
Coronary artery disease	1 (12.5%)
Fibroids	1 (12.5%)
Hepatitis B	2 (25.0%)
Hypothyroidism	2 (25.0%)
Parkinson’s disease	1 (12.5%)

IL, interleukin; TNF, tumour necrosis factor.

Rheumatoid arthritis (39.7%), systemic lupus erythematosus (23.8%), and spondyloarthritis (19.0%) were the leading rheumatologic indications, whereas multiple sclerosis (78.4%) predominated among neuroinflammatory disorders. Diffuse large B-cell lymphoma (51.2%) and chronic lymphocytic leukaemia (30.2%) were the most common malignancies. Table 2 summarises the diagnostic indications for biological therapies.

**Table 2:** Diagnostic indications for biologic therapy

Primary diagnosis	Disease	N = 204
Rheumatologic disorder (n = 63)	Rheumatoid arthritis	25 (39.7%)
	Systemic lupus erythematosus	15 (23.8%)
	Spondyloathritis	12 (19.0%)
	Sjogren’s syndrome	6 (9.5%)
	ANCA vasculitis	3 (4.8%)
	Psoriatic arthritis	2 (3.2%)
	Polymyositis	1 (1.6%)
	Behcet’s syndrome	1 (1.6%)
	Adult-onset Still’s disease	1 (1.6%)
	Antisynthetase syndrome	1 (1.6%)
	Stiff person syndrome	1 (1.6%)
	Antiphospholipid syndrome	1 (1.6%)
	Scleroderma	1 (1.6%)
Neuroinflammatory disorder (n = 37)	Multiple sclerosis	29 (78.4%)
	Neuromyelitis optica	2 (5.4%)
	Acute disseminated encephalomyelitis	1 (2.7%)
	Autoimmune epilepsy	1 (2.7%)
	Demyelinating disease	1 (2.7%)
	Neurosarcoidosis	1 (2.7%)
	Optic neuritis	1 (2.7%)
	Transverse myelitis	1 (2.7%)
Malignancy (n = 43)	Diffuse large B-cell lymphoma	22 (51.2%)
	Chronic lymphocytic leukaemia	13 (30.2%)
	Follicular lymphoma	3 (7.0%)
	Mantle cell lymphoma	2 (4.7%)
	Castleman’s disease	1 (2.3%)
	Marginal zone lymphoma	1 (2.3%)
	Small lymphocytic lymphoma	1 (2.3%)
Other primary diagnosis (n = 61)	Severe COVID-19	36 (59.0%)
	Myasthenia gravis	14 (23.0%)
	Focal segmental glomerulosclerosis	3 (4.9%)
	Immune thrombocytopenic purpura	2 (3.3%)
	Autoimmune haemolytic anaemia	1 (1.6%)
	Autoimmune thyroiditis	1 (1.6%)
	Rosai-Dorfman disease	1 (1.6%)
	Sclerosing mediastinitis	1 (1.6%)
	Takayasu arteritis	1 (1.6%)
	Ulcerative colitis	1 (1.6%)

ANCA; Anti-Neutrophil Cytoplasmic Antibodies.

### Biologic therapy characteristics

Three classes of biologic agents were identified. Anti-CD20 therapies were most used (71.6%), followed by IL-6 inhibitors (20.1%) and anti-TNF-α agents (9.3%). Rituximab accounted for the majority of anti-CD20 use. Median cumulative doses were highest for anti-CD20 agents (3000 mg, IQR, 2000-4116), whereas anti-TNF-α agents had the longest median duration of exposure (34.0 months, IQR, 15.0-65.0). Table 3 describes the biologic treatment characteristics.

**Table 3:** Biologic treatment characteristics of the study population

Parameter	N = 204	Duration of exposure in (mo), median [IQR]	Cumulative dose (mg), median [IQR]
Biologic agent class			
Anti-CD-20	146 (71.6%)	7.0 [1.0, 24.0]	3000.0 [2000.0, 4116.0]
IL-6 inhibitors	41 (20.1%)	0.0 [0.0, 0.0]	400.0 [400.0, 400.0]
Anti-TNF alpha Inhibitor	19 (9.3%)	34.0 [15.0, 65.0]	1750.0 [750.0, 3350.0]
Specific anti-CD-20 agent (n = 146)			
Rituximab	138 (94.5%)	7.0 [1.0, 23.0]	3000.0 [2000.0, 4200.0]
Ocrelizumab	11 (7.5%)	23.0 [19.0, 25.0]	2850.0 [1500.0, 3600.0]
Obinutuzumab	2 (1.4%)	3.0 [2.0, 4.0]	5000.0 [4000.0, 6000.0]
Specific IL-6 inhibitor agent (n = 41)			
Tocilizumab	41 (100.0%)	0.0 [0.0–0.0]^a^	400.0 [400.0, 400.0]
Specific anti-TNF alpha agent (n = 19)			
Golimumab	15 (78.9%)	40.0 [16.0, 67.0]	2000.0 [800.0, 3350.0]
Infliximab	4 (21.1%)	12.0 [2.0, 22.0]	912.5 [250.0, 2712.5]

IL, interleukin; TNF, tumour necrosis factor.

a The tociluzmab duration is reported as 0.0 months since majority of the patients,(n=36/41) were treated for severe COVID-19 with single dose.This acute ,non persistent pattern of administration is reflected in both the median and interquartile range(IQR),which is reported to have no period of continous exposure to the study in months.

### TB infection screening

Pretreatment screening for TB infection was performed in 69.1% of patients. Chest radiography was the most used modality (48.2%), followed by computed tomography (CT) chest (36.2%). IGRAs were performed in only 7.1% of screened patients, with a positivity rate of 30% (n = 3/10). No TST was performed. Among IGRA-positive individuals, 2 received chemoprophylaxis. No cases of TB disease occurred among patients diagnosed with TB infection during follow-up. Table 4 describes the TB infection screening profile.

**Table 4:** Tuberculosis infection screening profile of the study population

	N = 204
Any screening done	
No	63 (30.9%)
Yes	141 (69.1%)
Type of screening done (n = 141)	
CT chest	51 (36.2%)
PET – CT	21 (14.9%)
CTPA	2 (1.4%)
Chest X-ray	68 (48.2%)
IGRA	10 (7.1%)
TST	0 (0.0%)
Both IGRA + imaging	
IGRA + imaging (CXR)	4 (2.8%)
Results of IGRA (n = 10)	
Positive	3 (30.0%)
Negative	7 (70.0%)
Results chest X-ray (n = 68)	
Negative	68 (100.0%)
Results other tests^a^ (n = 74)	
Negative	74 (100.0%)
Screening per biologic class	
Anti-CD-20	89 (63.1%)
IL-6 inhibitors	37 (26.2%)
Anti-TNF alpha	9 (6.4%)
Screening per biologic agent	
Rituximab	83 (58.9%)
Ocrelizumab	4 (2.8%)
Obinutuzumab	2 (1.4%)
Tocilizumab	37 (26.2%)
Golimumab	8 (5.7%)
Infliximab	1 (0.7%)

CT, computed tomography; IGRAs, interferon-gamma release assays; IL, interleukin; TNF, tumour necrosis factor; TST, tuberculin skin test; PET, positron emission tomography; CTPA, computed tomography pulmonary angiogram; CXR, chest X-ray.

a CT Chest (n = 51); PET-CT (n = 21); CTPA (n = 2).

### TB disease characteristics

TB disease developed in 3 patients (1.5%; 95% CI, 0.3%-4.2%) during follow-up. Two cases were pulmonary and 1 extrapulmonary (genitourinary). All cases occurred in patients receiving rituximab. The median time to TB diagnosis after biologic initiation was 7.2 months. Two cases were microbiologically confirmed, whereas 1 was diagnosed clinically and radiologically.

Patients who developed TB disease had a median age of 59.7 years, and all were HIV-negative. Two had neuroinflammatory disorders (multiple sclerosis), and 1 had rheumatoid arthritis. TB infection screening was inconsistently performed; 1 patient had no screening at all, 1 had a normal chest X-ray only, and 1 had both a normal chest X-ray and CT chest used for screening (both negative at baseline). There were no TB cases during follow-up with anti-TNF-α inhibitor agents (golimumab and infliximab) or IL-6 inhibitor (tocilizumab) in this study population. Table 5 describes the details of the patients who developed TB disease.

**Table 5:** Details of patients who developed tuberculosis disease

	Patients (n = 3)		
Details	1	2	3
Age (y)	34	60	62
Sex	Female	Female	Male
Primary diagnosis	Multiple sclerosis	Multiple sclerosis	Rheumatoid arthritis
Biologic agent	Rituximab	Rituximab	Rituximab
TB infection screening	None	CXR only (normal)	CXR and CT chest (normal)
Time to diagnosis (mo)	30.7	7.2	5.2
Confirmation method	Clinical + CT findings	Bronchoalveolar lavage TB-PCR	Urine AFB stain
TB type	Pulmonary	Pulmonary	Genitourinary
Comorbidity	none	Hypothyroidism	Diabetes mellitus

TB, tuberculosis; CXR, chest X-ray; CT, computed tomography; AFB, acid- fast bacilli.

### *Comparative analysis of* TB disease and non-TB cases

There were no statistically significant differences between patients who developed TB disease and those who did not across most baseline variables. However, documented comorbidities were more frequent among TB cases, including diabetes mellitus and hypothyroidism (66.7% vs 10.0%, *P* = .031). Patients with TB disease were older, with a median age of 59.7 years [IQR, 33.9-61.8], vs non-TB median age of 45.6 years [IQR, 36.4-60.1], though this was not statistically significant (*P* = .657). Table 6 summarises the comparisons.

**Table 6:** Comparison of patients with and without tuberculosis disease

Characteristic	Non-TB (n = 201)	TB disease (n = 3)	*P* Value
Age (y), median [IQR]	45.6 [36.4, 60.1]	59.7 [33.9, 61.8]	.657
Sex, female n (%)	119 (59.2%)	2 (66.7%)	1.000
Primary diagnosis category, n (%)			
Rheumatologic	62 (30.8%)	1 (33.3%)	1.000
Neuroinflammatory	35 (17.4%)	2 (66.7%)	.085
Malignancy	43 (21.4%)	0 (0.0%)	1.000
Other	61 (30.3%)	0 (0.0%)	.556
Biologic class, n (%)			
Anti-CD-20	143 (71.1%)	3 (100.0%)	.560
IL-6 inhibitor Anti-TNF alpha	41 (20.4%)	0 (0.0%)	1.000
	19 (9.5%)	0 (0.0%)	1.000
HIV status, positive n (%)	7 (3.5%)	0 (0.0%)	1.000
Documented comorbidity, n (%)^a^	20 (10.0%)	2 (66.7%)^b^	.031
TB infection screening, n (%)	139 (69.2%)	2 (66.7%)	1.000

IL, interleukin; TB, tuberculosis; TNF, tumour necrosis factor.

a (Non-TB), hypertension (n = 11), diabetes mellitus (n = 9), and chronic kidney disease (n = 5).

b (TB disease), diabetes mellitus (n = 1) and hypothyroidism (n = 1).

## DISCUSSION

This study provides the first novel real-world data on TB disease outcomes among patients receiving biologic agents in a high-burden setting of Kenya and, more broadly, East Africa. Evidence from SSA on outcomes of TB in patients treated with biologic agents remains limited, although the region has a disproportionately high caseload of TB. Most prior studies have originated from South Africa and North Africa and have focused largely on rheumatologic populations treated with TNF-α inhibitors [[20], [21], [22], [23]]. Our findings extend the literature by describing TB disease proportion, TB infection screening practices, and clinical characteristics across a heterogeneous cohort in Kenya.

The study population was predominantly middle-aged with a median age of 45.8 years and female predominance (59.3%). This population trend is both widely consistent with published patterns in international biologic cohorts, although some variability exists across regions [9,20,22,23]. Unlike most published cohorts, biologic use in this setting was not restricted to rheumatologic diseases; substantial proportions of patients were treated for neuroinflammatory disorders, haematologic malignancies, and other immune-mediated conditions. This broader indication profile reflects evolving prescribing patterns and therefore the need to consider TB risk across multiple specialties.

A key finding was the predominance of anti-CD20 therapy (71.6%), particularly rituximab, in contrast to global and regional registries in which anti-TNF-α agents were most commonly used [20,[22], [23], [24], [25]]. This difference has important implications for TB risk. Although TNF-α inhibitors are classically associated with TB reactivation due to their role in granuloma maintenance [14,16], emerging evidence suggests that non-TNF-α biologics, including B-cell depleting agents, may also confer clinically relevant risk, particularly in endemic settings [21]. Notably, all TB cases in our cohort occurred among patients receiving rituximab, supporting the need to broaden TB-risk assessment beyond TNF-α inhibitors alone.

In Kenya, there are no standardised national TB infection screening and treatment protocols for adults receiving biologic therapy. Existing global recommendations advise intensive TB infection screening followed by prophylaxis, where necessary [13]. Pretreatment TB infection screening in the study population was suboptimal, with approximately one-third of patients (30.9%) not screened. Among those screened (69.1%), reliance on chest radiography and other imaging modalities such as CT chest was common, whereas use of immunologic testing was limited to IGRA usage (7.1%). The low uptake of IGRA and absence of TST use highlight gaps in adherence to international recommendations, likely reflecting resource and availability constraints [26]. Importantly, although overall IGRA usage was low, the positivity rate of 30% notably exceeded the 21.8% prevalence in the general adult population [3], suggesting a substantial burden of TB infection in this population, likely compounded by comorbidities such as diabetes mellitus and immunosuppression. Notably, baseline imaging was negative in all patients who subsequently developed TB disease, emphasising the limited sensitivity of radiologic screening alone, particularly in immunocompromised hosts [13]. The occurrence of TB disease despite negative baseline screening imaging raises the possibility of false-negative screening, subclinical infection, or *de novo* TB acquisition during therapy, all of which are plausible in high-burden settings.

In this cohort, TB disease developed in 1.5% of the patients, corresponding to an estimated incidence rate of about 245 per 100,000 person-years. This was comparatively lower than reported rates in other African biologic cohorts, including South African and Moroccan studies that had rates of 1240–2227 per 100,000 person-years and 1700 per 100,000 person-years, respectively [[21], [22], [23]]. These observed differences may be attributable to the biologic exposure profile, with lower use of anti-TNF-α agents in our cohort, as well as differences in background TB epidemiology. All TB cases occurred with the anti-CD20 agent, rituximab, with no cases observed among those treated with anti-TNF-α agents or IL-6 inhibitors. Although the small number of events precludes definitive conclusions, this pattern aligns with findings from the Western Cape cohort, where non-TNF biologics, particularly rituximab, accounted for a substantial proportion of TB cases [21]. These observations challenge the traditional perception supported by robust evidence that TB risk is lowest with B-cell-depleting therapies [12]. However, this observation should also be interpreted with caution rather than as evidence of a causal, agent-specific effect that is difficult to attribute definitively to anti-CD20 therapy while considering confounders like burden of comorbidity, immunosuppression from the underlying disease itself, and associated gaps in screening at baseline, particularly given that all the 3 cases had negative or absent imaging, and none underwent IGRA testing. The possibility of pre-existing subclinical TB not detected on screening also exists rather than true post-treatment reactivation or new acquisition. For context, applying Kenya’s national TB incidence estimate (207 per 100,000 population) [26] to this cohort’s approximately 245 per 100,000 person-years would predict roughly 2 to 3 incident cases even in the complete absence of biologic exposure, suggesting that the observed 3 cases are closely aligned with, rather than clearly more than, the background risk. Taken together, these findings should be read as hypothesis-generating rather than confirmatory of a rituximab-specific TB signal, and they highlight the need for prospective, adequately powered studies with systematic baseline and follow-up screening across all biologic classes before firm causal inferences about agent-specific TB risk can be drawn.

Data from non–biologic-exposed populations using immune suppression such as corticosteroids and conventional synthetic disease modifying anti-rheumatic drugs including methotrexate and leflunomide with comparable comorbidity profiles in TB-endemic settings are scarce, but studies consistently show TB incidence above national background rates in the presence of similar risk factors (diabetes, malignancy, and chronic immunosuppressive states) even at baseline and higher risk with immunosuppressive therapy, especially in combination of corticosteroid with methotrexate and leflunomide, whereas sulfasalazine and hydroxychloroquine consistently showed the lowest risk [12,27], suggesting that much of the excess risk observed here may reflect the underlying disease burden.

The absence of TB disease among IL-6 inhibitor-treated patients in our cohort, most of whom received single-dose tocilizumab for severe COVID-19 (87.8%), is consistent with existing evidence distinguishing short-term from chronic exposure risk. Short-course IL-6 inhibition has been shown to carry a favourable safety profile and negligible risk of secondary infections or TB reactivation [28] compared with chronic, repeated-dose regimens used in rheumatological conditions, with a reported small but significant increase in infection risk with combination therapy [29].

The median time to TB diagnosis was 7.2 months following biologic initiation, consistent with the report of rituximab-specific data from a high-burden setting with a median time of 7.1 months [21], highlighting a period of increased vulnerability early in therapy, although risk persists beyond this timeframe. Both pulmonary (66.7%) and extrapulmonary (33.3%) disease were observed, reflecting the spectrum of TB in immunocompromised populations, with similar findings noted in Western Cape data [21].

Although no statistically significant predictors of TB were identified, likely due to the small number of events, patients who developed TB disease were older and more likely to have comorbidities. The limited documentation of key risk factors, including prior TB history and HIV status, further constrained risk stratification. Comorbid diabetes mellitus was noted in 1 of the TB cases, and comorbidities such as diabetes mellitus and a history of previous TB played a role in increased TB risk in South African Biologics Registry and Moroccan studies [22,23].

This study had several limitations. It was conducted at a single private tertiary centre, which may limit generalisability to broader populations, particularly in public healthcare settings where most TB care is provided. Although this cohort is the only Kenyan study of patients treated with biologic agents assessed for the outcome of TB disease, the small sample size (n = 204) and low number of TB events (n = 3) compared with other studies and biologic registries [21,23,30] limited the statistical power of subgroup analysis and risk factor evaluation. Additionally, the retrospective design resulted in incomplete data capture for important variables, including HIV status, TB infection screening details, and prior TB exposure. The study inclusion criteria to only include patients initiated on biologic therapy on or after January 1, 2018, with at least 3 years of follow-up, resulted in the exclusion of a substantial proportion of the source population (735/939 patients). Although these criteria were necessary to standardise the cohort’s index date and to ensure consistent baseline data capture and follow-up, these likely excluded prevalent long-term biologic users who could have added further person-time and TB events, potentially underestimating true TB incidence and reducing power for subgroup analyses. Furthermore, the study only sought to look at the proportion of TB disease rather than TB infection in this population.

Despite these limitations, our study highlights important clinical implications in the face of growing use of biologic agents worldwide. TB risk in patients receiving biologic therapy extends beyond TNF-α inhibitors and remains clinically relevant in high-TB burden settings. Strengthening TB infection screening practices, particularly increasing access to and use of immunologic testing alongside implementing standardised protocols for screening, prophylaxis, and follow-up, is essential. Furthermore, the establishment of national or regional biologic therapy registries would be valuable in facilitating improved surveillance and risk assessment of patients receiving biologic therapies and supporting safer use of biologic therapies in TB-endemic settings.

Further prospective multicentre studies with larger and more diverse populations, including public sector hospitals that are adequately powered with systematic baseline and follow-up screening across all biologic classes, are recommended before firm causal inferences about agent-specific TB risk can be drawn, to better define TB risk and inform context-specific screening and prevention strategies in TB-endemic regions.

## CRediT authorship contribution statement

**Subo Hassan Marsa:** Writing – review & editing, Writing – original draft, Visualization, Validation, Project administration, Methodology, Investigation, Funding acquisition, Formal analysis, Data curation, Conceptualization. **Fredrick Otieno Owuor:** Writing – review & editing, Writing – original draft, Visualization, Validation, Supervision, Resources, Project administration, Methodology, Formal analysis, Data curation, Conceptualization. **Jasmit Shah:** Writing – review & editing, Supervision, Software, Methodology, Formal analysis, Data curation, Conceptualization. **Felix Riunga Mutuma:** Writing – review & editing, Writing – original draft, Visualization, Validation, Supervision, Resources, Project administration, Methodology, Formal analysis, Data curation, Conceptualization.

## Competing interests

All authors declare they have no competing interests.
